# Inetetamab for injection in combination with vinorelbine weekly or every three weeks in HER2-positive metastatic breast cancer: A multicenter, randomized, phase II clinical trial

**DOI:** 10.1515/jtim-2024-0022

**Published:** 2024-11-06

**Authors:** Xiying Shao, Ning Xie, Zhanhong Chen, Xinshuai Wang, Wenming Cao, Yabing Zheng, Hua Yang, Jian Huang, Shaoping Chen, Lu Gan, Xiuli Yang, Yuru Chen, Quchang Ouyang, Xiaojia Wang

**Affiliations:** Department of Breast Medical Oncology, Zhejiang Cancer Hospital, Hangzhou 310022, Zhejiang Province, China; Medical Department of Breast Cancer, Hunan Cancer Hospital, Changsha 410013, Hunan Province, China; The 1st Affiliated Hospital of Henan University of Science and Technology, Luoyang 471003, Henan Province, China; Department of Medical Oncology, Affiliated Hospital of Hebei University; Hebei Key Laboratory of Cancer Radiotherapy and Chemotherapy, Shijiazhuang 071000, Hebei Province, China; Department of Oncology, Dongying People’s Hospital, Dongying 257091, Shandong Province, China; The First Hospital of Chongqing Medical University Affiliated Hospital. Medical Oncology, Chongqing 400016, China; The First Affiliated Hospital of Nanyang Medical College, Nanyang 473007, Henan Province, China; Shandong Provincial Hospital Heze Hospital (Heze Municipal Hospital) Oncology Department, Heze 274000, Shandong Province, China

**Keywords:** metastatic breast cancer, pharmacokinetics, safety, efficacy, immunogenicity

## Abstract

**Objective:**

We aimed to investigate the pharmacokinetics, safety, efficacy, and immunogenicity of different dosing regimens (weekly and every three weeks) of inetetamab in combination with vinorelbine in human epidermal growth factor receptor 2 (HER2)+ patients with metastatic breast cancer who had received one or more chemotherapy regimens.

**Methods:**

HER2+ patients with metastatic breast cancer who had received one or more chemotherapy regimens were included. Eligible patients received inetetamab administered weekly or every three weeks in combination with vinorelbine injection chemotherapy. Pharmacokinetics, safety, efficacy, and immunogenicity were compared between the groups.

**Results:**

Sixty HER2+ patients were randomized into a single-week administration group ( *n* = 29) and a three-week administration group ( *n* = 31). After the final dose in the single-week administration group and the three-week administration group, the mean C_max_ values were 79.773 μg/mL and 146.083 μg/mL; the mean C_min_ values were 30.227 μg/mL and 11.926 μg/mL; the mean AUC_tau_ values were 7328.443 μg·h/mL and 22647.101 μg·h/mL; and the mean C_av_ values were 43.622 μg/ mL and 44.935 μg/mL, respectively. The best overall response (BOR) rates at 24 weeks and unconfirmed BOR rates at 24 weeks were both 40.7% in the single-week dosing group and 40.7% in the three-week dosing group, and the 24-week confirmed disease control rates (DCRs) were 88.9% and 81.5%, respectively. The incidence of adverse events (AEs) was generally consistent across all levels.

**Conclusion:**

There were slight differences in the mean C_max_, C_min_, AUC_tau_ and C_av_ between the three-week dosing group and the single-week dosing group, and the mean steady-state concentrations of C_av_ were comparable; however, there were no differences in efficacy, safety or immunogenicity between the two groups.

## Introduction

Breast cancer is a global health concern with a significant impact on the well-being of women.^[1]^ A range of modifiable and non-modifiable risk factors have been established as being associated with an increased risk of developing breast cancer.^[2,3]^ The human epidermal growth factor receptor (HER) family is a class of transmembrane proteins with tyrosine protein kinase activity that regulates cell growth and differentiation.^[4,5]^ The general structure of the HER family of transmembrane receptors consists of an extracellular ligand-binding region, a transmembrane structural region, and an intracellular tyrosine kinase region.^[6,7]^ HER2+ breast cancer is highly dependent on the sustained overexpression of HER2 oncogene proteins and their complex downstream signaling pathways,^[8]^ and 20%–30% of breast cancer patients are clinically diagnosed with this type of cancer.^[9,10]^ For HER2+ breast cancer patients, targeted therapy for HER2 has been established as the standard of care, and the use of specific HER2-targeted drugs has been shown to be effective, significantly increasing progression-free survival (PFS) and overall survival (OS).^[11,12,13,14]^

The standard first-line treatment for HER2+ advanced breast cancer without prior anti-HER2 therapy is chemotherapy combined with trastuzumab-based targeted therapy, which can significantly prolong the OS of patients.^[11,15,16,17]^ Trastuzumab, the initial humanized monoclonal antibody that targets HER2, has substantially improved the prognosis of individuals with HER2+ breast cancer and has altered diagnostic and therapeutic approaches for breast cancer, representing a significant advancement in targeted therapy for this disease.^[18,19]^ In addition, novel anti-HER2 therapeutic agents, such as the tyrosine kinase inhibitor pyrotinib, and antibody-drug conjugates (ADCs), including T-DM1 and T-DXd, have continuously emerged, further improving the prognosis of HER2+ breast cancer patients.^[20,21,22,23]^ However, studies have shown that there is a significant difference in the utilization rate of trastuzumab due to the uneven allocation of health care resources in China.^[24]^ Moreover, while trastuzumab administration can considerably prolong patient survival, its utilization during the early stages of breast cancer may impact the efficacy of subsequent metastasis treatment.^[25]^ There is clearly room for improvement in terms of the limited selection and exorbitant cost associated with anti-HER2 monoclonal antibodies, thereby highlighting a substantial unmet demand in treatment. Consequently, the Chinese government actively promotes and endorses autonomous exploration and creation of innovative drugs to address the practical needs of individuals with breast cancer.

Inetetamab, a product developed by Sunshine Guojian Pharmaceutical (Shanghai) Co., Ltd., is the result of refining the process of expressing the recombinant humanized anti-HER2 monoclonal antibody and was built upon prior foreign research endeavors.^[26]^ Like trastuzumab, inetetamab acts on the extracellular part of the HER2 receptor, blocking the activation of intracellular tyrosine kinases and blocking the activation of human epidermal growth factor to HER2 by attaching itself to HER2, thus blocking the growth of cancer cells. Compared with trastuzumab, inetetamab, a newly developed drug, has a late-mover advantage, with two antigen-binding fragments (Fabs), consisting of 214 amino acids each. However, inetetamab diverges from trastuzumab, as its fragment crystallizable (Fc) has undergone amino acid modifications, rendering it distinct from biosimilars. This Fc modification imbues inetetamab with a greater antibody-dependent cell-mediated cytotoxic effect (ADCC) compared to trastuzumab.^[27]^ The culmination of preclinical investigations and phase I, II, and III clinical trials involving patients with HER2+ breast cancer has demonstrated that the combination of inetetamab and vinorelbine chemotherapy is superior regarding efficacy and safety.^[28]^ The HOPES study showed that the median progression-free survival (mPFS) for the first-line treatment subgroup was 11.2 months with an objective response rate (ORR) of 61.5% for the HER2+ MBC patients in China with inetetamab in combination with chemotherapy, and the mPFS for the multiline treatment group was 9.2 months, and the ORR was still as high as 46.7%.^[28]^ The product underwent approval from the National Medical Products Administration (NMPA) in July 2004 for clinical research (Approval No. 2004 L02352) and successfully completed phase I to III clinical trials, leading to its marketing approval in June 2020. Inetetamab, which has only been approved in a single country, lacks sufficient clinical study data to explore delivery methods to achieve the best clinical benefit compared with trastuzumab, which has been mature and used globally for many years. For cancer patients in developing countries, a heavy economic burden is often the greatest factor in the nonmedical interruption of treatment. To reduce the financial burden on patients, this study aimed to explore the potential cost reduction of drugs by extending the dosing interval while maintaining the same efficacy and safety. Notably, there are currently no published studies on the dosing intervals of inetetamab. Specifically, this study aimed to explore the differences in the various inetetamab administration modes, focusing on the pharmacokinetic profile of inetetamab when combined with vinorelbine injection in both single- and every three-week dosing regimens, as well as to compare the safety, efficacy, and immunogenicity.

## Methods

### Patients

This multicenter, randomized, phase II clinical study evaluated the pharmacokinetics, efficacy, and safety of inetetamab in combination with vinorelbine administered weekly or every three weeks for the treatment of patients with HER2+ metastatic breast cancer. This study was registered at ClinicalTrials.gov (NCT05131841).

Eligible patients are adults (18–70 years) with histologically or pathologically confirmed unresectable metastatic breast cancer who had received one or more chemotherapy regimens, have a body mass index (BMI) ranging from 19.0–28.0, and are HER2-positive, defined as “+ ++” by immunohistochemistry (IHC) or fluorescence in situ hybridization (FISH) of “+” (according to the Breast Cancer HER2 Detection Guidelines 2019 Edition). An Eastern Cooperative Oncology Group (ECOG) physical status score of 0 or 1 and at least one measurable lesion according to the Response Evaluation Criteria in Solid Tumors, version 1.1 (RECIST 1.1), were also required. The key exclusion criteria included patients those whose first treatment with the study drug was less than 28 days or less than 5 half-lives from their most recent dose of another drug that may have affected the pharmacokinetic outcome of the study drug, as well as the use of a monoclonal antibody analog within the previous 6 months, uncontrolled disease or active infection, a history of severe cardiac disease, cardiac risk factors or uncontrolled cardiac arrhythmia, active or recent coronary artery events, andpregnancy and/or lactation. The study included a 28-day screening period and a 24-week treatment period. After the completion of treatment, the subjects completed end-of-study visits at D169 ± 3 days. The protocol was approved by the relevant institutional review boards/independent ethics committees of Zhejiang Hospital (IRB-2020-224). The study strictly followed the principles of the International Conference on Harmonization, Good Clinical Practice guidelines, the Declaration of Helsinki, and local laws and regulations. Written informed consent was obtained prior to all study procedures.

### Study design and treatment

**Figure 1:**
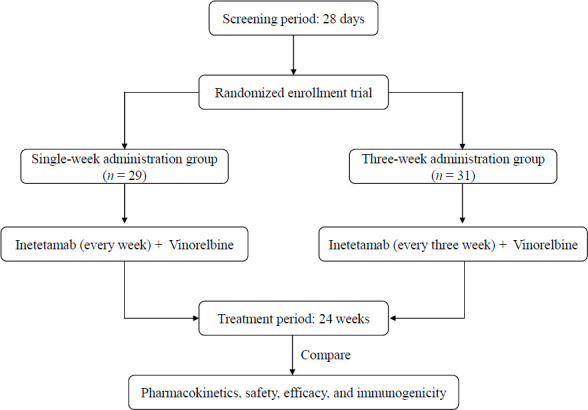
Single and three-week dosing schedules of inetetamab.

This clinical trial consisted of a screening phase (V1 visit: from D28 to D-1), a treatment phase (V2-V10 visits: from D1 to D169 ± 3), a safety follow-up (28 ± 3 days after the last dose), and a survival follow-up (every 12 weeks ± 7), which was considered complete when the subjects completed the end-of-study visit on D169 ± 3 days. Patients were randomized into two cohorts: a single-weekly dosing group and a three-weekly dosing group (Figure 1). The specific dosing regimens were as follows.

### Inetetamab

Single-weekly dosing: inetetamab was administered by intravenous infusion once weekly. The first dose of 4 mg/kg was administered over a period of no less than 90 min, followed by 2 mg/kg weekly and subsequent intravenous drip over a period of no less than 30 min if well tolerated by the patient at the time of the first intravenous drip.

Three-weekly dosing: inetetamab was administered *via* intravenous drip every three weeks. The first dose was 8 mg/kg, and the first intravenous drip was no less than 90 min, followed by 6 mg/kg every three weeks, with subsequent infusions completed in 30–90 mins if well tolerated by the patient at the time of the first intravenous drip.

The left ventricular ejection fraction (LVEF) was measured prior to initiating treatment with this product, and was routinely monitored during treatment. Treatment was suspended for at least 3 weeks if there was a > 10% absolute decrease in LVEF from pretreatment values and if the absolute LVEF decreased to less than 50%. If the LVEF rebounded to ≥50% or if the absolute decrease in LVEF was ≤10% within 3 weeks, therapy with this product was resumed; if there was no improvement or further decrease in LVEF or if clinically significant congestive heart failure occurred, therapy with this product was discontinued.

### Vinorelbine injection

All patients received vinorelbine injection (25 mg/m^2^, days 1 and 8, intravenously, every 21 days, *i.e*., 1 cycle of 3 weeks, on the same day as the inetetamab infusion). The treatment period lasted 24 weeks and continued until disease progression, unacceptable toxicity, withdrawal of consent, loss to follow-up, or study termination.

### Endpoints

The primary endpoints were the pharmacokinetic parameters C_max_, C_min_, AUC_0-t_, and AUC_tau_ after the last dose; the secondary observations were C_max_, C_min_, AUC_0-t_, AUC_tau_, and T_max_ after the first dose, the 3rd dose in the three-weekly dosing group, and the 7th dose in the single-weekly dosing group. The key secondary endpoints included best overall response (BOR), disease control rate (DCR), adverse drug reactions (ADRs) and adverse events (AEs).

### Assessments

#### Pharmacokinetics

Upper limb venous blood was collected from the patients after the first dose, the 3rd dose in the three-weekly dosing group, the 7th dose in the single-weekly dosing group and the last dose, and the concentration of the drug in the serum was determined *via* ELISA to obtain the pharmacokinetic parameters of the drug.

#### Anti-tumor efficacy

The antitumor efficacy of each group after drug administration was evaluated by imaging examinations and other examination results. The BOR, DCR, and OS after drug administration were evaluated with reference to RECIST V1.1. Imaging and baseline assessments wereperformed once during the screening phase, and imaging and efficacy assessments were performed every 6 weeks during the treatment phase. Imaging examinations were required at the end-of-study visit or early termination of the treatment visit and could not be repeated if imaging examinations were available within 4 weeks. BOR defined as percentage of participants with best overall (objective) response based assessment of confirmed complete response (CR) or confirmed partial response (PR). Unconfirmed BOR defined as percentage of participants with best overall (objective) response based assessment of unconfirmed CR or unconfirmed PR. If a patient achieved a first CR or PR on tumor assessment at week 24, they continued to receive 6 weeks of inetetamab monotherapy with confirmatory imaging evaluation, with the corresponding end-of-study visit and safety follow-up were postponed.

#### Safety

AEs, SAEs, laboratory tests, vital signs, physical examination, 12-lead electrocardiogram (12-ECG), and echocardiograms were evaluated during the trial phase. The severity of the AEs was assessed with reference to the National Cancer Institute Common Terminology Criteria for Adverse Events (NCI CTCAE, version 5.0).

#### Immunogenicity

Patient blood samples were assayed for the incidence and titer of the anti-drug antibody (ADA) associated with the serum levels of the test drug, as well as for the incidence of neutralizing antibodies. The ADA assay was performed *via* the validated bridging-ELISA method, and ADA-positive samples were reanalyzed for titer and neutralizing antibody (Nab).

### Statistical analysis

The pharmacokinetic parameters were calculated according to the nonatrial model *via* WinNonlin 8.2 (Certara, US). Blood concentration-time curves and mean blood concentration-time curves were plotted for each patient on the basis of the blood concentration-time data measured in the trial. All the AEs were standardized *via* Medical Dictionary for Regulatory Activities (MedDRA) codes, and summary statistics were performed according to the system-organ classification (SOC) and preferred term (PT) in the MedDRA codes and the severity of the AEs. For BOR and DCR, the Clopper Pearson method was used to estimate and provide the corresponding 95% CI. For OS, the Kaplan Meier method was used to estimate the median value and its 95% CI, and survival curves were plotted. The difference was significant if the *P* value was < 0.05.

## Results

### Patient characteristics

In total, 60 patients were successfully enrolled and treated with the drug: 29 in the single-weekly dosing group and 31 in the three-weekly dosing group. Fifty-four patients were included in the full analysis set (FAS) (27 in the singleweekly dosing group and 27 in the three-weekly dosing group), and 57 patients were included in the safety set (SS), pharmacokinetics (PK) concentration set (PKCS), and PK parameter set (PKPS) (27 in the single-weekly dosing group and 30 in the three-weekly dosing group for each of the analysis sets). Demographic analysis of the 57 patients included in the SS set revealed that the median age was 55.0 years (range: 26–67), the median age in the single-weekly dosing group was 53.0 years (range: 26–65), and the median age in the three-weekly dosing group was 56.0 years (range: 28–67)(Table 1). The patients enrolled were all female and were HER2+ metastatic breast cancer patients who had received one or more chemotherapy regimens. There were 47 patients (82.5%) with a previous history of breast cancer surgery, 25 patients (92.6%) in the single-weekly dosing group and 22 patients (73.3%) in the three-weekly dosing group. There were 57 patients (100.0%) with a previous history of chemotherapy. There were 30 patients (52.6%) with a previous history of radiotherapy, 12 patients (44.4%) in the single-weekly dosing group and 18 patients (60.0%) in the three-weekly dosing group. There were 16 patients with second-line treatment at enrollment and 41 patients with more than second-line treatment.

**Table 1:** Population statistics (SS)

Items	Single-week administration group (*n* = 27)	Three-week administration group (*n* = 30)	Statistics	*P*
Age			-1.452	0.152
Mean (SD)	51.0 (9.0)	54.4 (8.9)		
Median (Q1, Q3)	53.0 (46.0, 56.0)	56.0 (50.0, 61.0)		
Min, Max	26.0, 65.0	28.0, 67.0		
Nation				0.239
Ethnic Han	27 (100.0)	27 (90.0)		
Others	0	3 (10.0)		
Height (cm)			-1.323	0.192
Mean (SD)	156.7 (3.7)	158.6 (6.6)		
Median (Q1, Q3)	156.0 (155.0, 160.0)	158.0 (155.0, 162.0)		
Min, Max	150.0, 165.0	145.0, 175.0		
Weight (kg)			-0.539	0.592
Mean (SD)	57.7 (7.7)	58.7 (7.1)		
Median (Q1, Q3)	58.0 (52.0, 64.4)	59.0 (53.0, 65.0)		
Min, Max	43.0, 71.0	47.0, 70.0		
BMI (kg/m)			0.184	0.855
Mean (SD)	23.5 (2.9)	23.4 (2.5)		
Median (Q1, Q3)	23.4 (21.4, 26.1)	22.5 (21.5, 25.6)		
Min, Max	19.1, 27.9	19.5, 27.8		

### Pharmacokinetics

**Figure 2:**
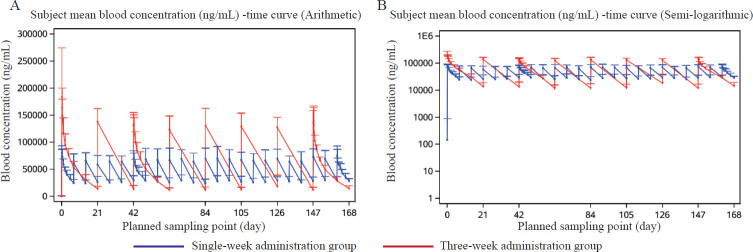
Mean serum concentration-time curve after infusion of inetetamab in the one-week and three-week administration groups.

After the first administration, the median T_max_ values of the patients in each dosing group were similar (Table 2 and Table 3), at 2.40 h and 2.09 h for the single-weekly and three-weekly dosing groups, respectively. The mean C_max_ values were 82.83 μg/mL and 192.63 μg/mL (Figure 2), respectively. The mean AUC0t was 6990.58 μg·h/mL and 23965.45 μg·h/mL, respectively. The mean AUC_0-inf_ values were 5629.80 μg·h/mL and 27744.15 μg·h/mL, and the mean clearance rate (CL) values were 0.72 mL/h/kg and 0.30 mL/h/kg for patients in the single-weekly and threeweekly dosing groups, respectively. The mean V_d_ was 74.42 mL/kg, and the mean V_d_ was 67.30 mL/kg. The mean t_½_ values were 72.83 h (3.03 days) and 159.96 h (6.67 days), respectively.

**Table 2:** Descriptive statistics of the inetetamab pharmacokinetic parameters in the serum after a single-week administration of inetetamab-first, seventh and last administration (PKPS)

Group	C_max_ (μg/mL)	C_min_ (μg/mL)	T_max_^a^ (h)	C_av_ (μg/mL)	AUC_tau_ (μg·h/mL)	AUC_tau_ (μg·h/mL)	MRT_0-t_ (h)	AUC_0-inf_ (μg·h/mL)	CL (mL/h/ kg)	V_d_ (mL/kg)	t_½_ (h)	λz (1/h)
First administration (*n* = 27)	82.83± 15.04 (18.2%)	0.14 ± 0.75 (519.6%)	2.4 (1.48, 25.60)	41.31 ± 7.47 (18.1%)	6990.58 ± 1353.46 (19.4%)	6939.56 ± 1254.57 (18.1%)	68.33 ± 4.75 (6.9%)	5629.80 ± 888.00^b^ (15.8%)	0.72 ± 0.11^b^ (15.8%)	74.42 ± 3.01^b^ (4%)	72.83 ± 14.39^b^ (19.8%)	0.010 ± 0.002^b^ (19.8%)
7th administration (*n* = 27)	74.10 ± 14.34^c^ (19.4%)	29.33 ± 8.54^c^ (29.1%)	1.13^c^ (0.58, 24.02)	43.07 ± 9.40^c^ (21.8%)	6935.42 ± 1501.22^c^ (21.6%)	7236.27 ± 1579.60^c^ (21.8%)	68.51 ± 5.98^c^ (8.7%)	NA	NA	NA	NA	NA
Last administration (*n* = 27)	79.77 ± 20.07 (25.2%)	30.23 ± 5.69^d^ (18.8%)	3.65^d^ (0.60, 41.78)	43.62 ± 5.57^d^ (12.8%)	7225.04 ± 1089.95^d^ (15.1%)	7328.44 ± 935.64^d^ (12.8)	69.96 ± 6.40^d^ (9.1%)	NA	NA	NA	NA	NA

NA: Because AUC__%Extrap_ exceeded 20% or some subjects did not sample the dense sampling cycle due to fewer phase elimination sampling points, relevant parameters such as AUC_0-inf_, CL, V_d_, t_½_ and λz could not be accurately calculated. The list is for reference only and is not included in the descriptive statistical analysis. a: T_max_ is displayed according to the median value (minimum value, maximum value). The summary results of the remaining parameters are displayed according to the arithmetic mean ± standard deviation (coefficient of variation %). b: *n* = 2; c: *n* = 23; d: *n* = 11.

**Table 3:** Descriptive statistics of the inetetamab pharmacokinetic parameters in the serum after three weeks of inetetamab administration—the first, third and last administration (PKPS)

Group	C_max_ (μg/mL)	C_min_ (μg/mL)	T_max_^a^ (h)	C_av_ (μg/mL)	AUC_0-t_ (μg·h/mL)	AUC_tau_ (μg·h/mL)	MRT_o–t_ (h)	AUC_0-inf_ (μg·h/mL)	CL (mL/h/kg)	V_d_ (mL/kg)	t_½_ (h)	λz (1/h)
First administration (*n* = 29)	192.63 ± 89.38 (46.4%)	0	2.09 (1.53, 9.67)	48.29 ± 9.26 (19.2%)	23965.45 5599.41 (23.4%)	± 24336.68 4668.57 (19.2%)	± 156.16 34.43 (22%)	± 27744.15 4855.52^b^ (17.5%)	± 0.30 ± 0.06^b^ (21.4%)	67.30 ± 11.82^b^ (17.6%)	159.96 ± 30.76^b^ (19.2%)	0.005 ± 0.001^b^ (26.5%)
3th administration (*n* = 29)	137.57 ± 24.04^c^ (17.5%)	13.27 ± 6.55^c^ (49.4%)	3.43 (0.55, 8.78)	43.76 ± 7.19^c^ (16.4%)	22181.27 3710.36^c^ (16.7%)	± 22053.35 3621.49^c^ (16.4%)	± 167.25 14.10^c^ (8.4%)	± 24885.28 4408.94^d^ (17.7%)	± 0.25 ± 0.05^d^ (19.5%)	55.20 ± 8.20^d^ (14.9%)	156.64 ± 24.76^d^ (15.8%)	0.005 ± 0.001^d^ (19%)
Last administration (*n* = 29)	146.08 ± 29.47^e^ (20.2%)	11.93 ± 4.73^e^ (39.7%)	3.73^e^ (0.77, 8.68)	44.94± 6.39^e^ (14.2%)	22594.82 3235.22^e^ (14.3%)	± 22647.10 3218.82^e^ (14.2%)	± 167.62 15.35^e^ (9.2%)	± 25388.90 4486.01^f^ (17.7%)	± 0.24 ± 0.04^f^ (18.2%)	59.31 ± 9.57^f^ (16.1%)	174.30 ± 40.42^f^ (23.2%)	0.004 ± 0.001^f^ (28.7%)

After the 7th dose in the single-weekly dosing group and the 3rd dose in the three-weekly dosing group, the mean C_max_ values were 74.10 μg/mL and 137.57 μg/mL; the mean C_min_ values were 29.33 μg/mL and 13.27 μg/mL; the mean AUC_tau_ values were 7236.27 μg·h/mL and 22053.35 μg·h/mL; and the mean Cav values were 43.07 μg/mL and 43.76 μg/mL, respectively.

Following the administration of the last dose in the singleweekly and three-weekly dosing groups, the mean C_max_ values were 79.77 μg/mL and 146.08 μg/mL, respectively; the mean C_min_ values were 30.23 μg/mL and 11.93 μg/mL; the mean AUC_tau_ values were 7328.44 μg·h/mL and 22647.10 μg·h/mL, respectively; and the mean Cav was 43.62 μg/mL for the single-weekly group and 44.94 μg/mL for the three-weekly group.

**Figure 3:**
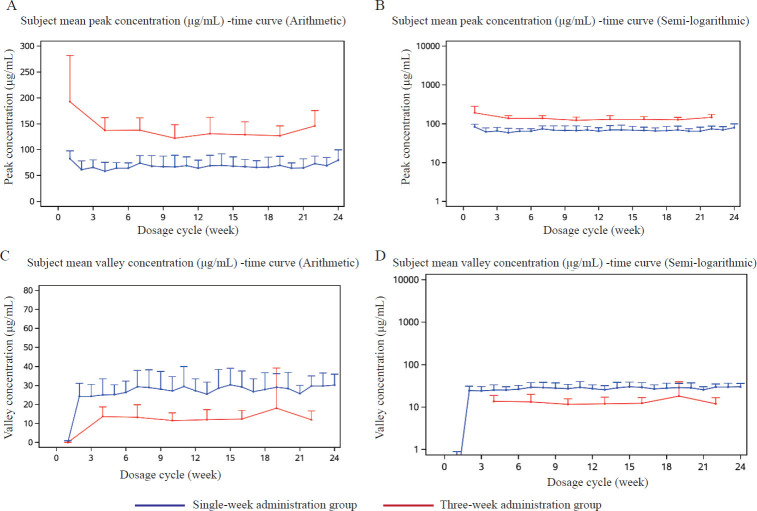
Average serum concentration-time curves of peak concentration and trough concentration after inetetamab infusion in single - and three-week groups.

After the third and final doses in the three-weekly dosing group, the mean CL values were 0.25 mL/h·kg and 0.24 mL/h·kg, respectively; the mean V_d_ was 55.20 mL/kg for the single-weekly group and 59.31 mL/kg for the threeweekly group; and the mean t_½_ was 156.64 h (equivalent to 6.53 days) for the single-weekly group and 174.30 h (equivalent to 7.26 days) for the three-weekly group. The accumulation ratios of C_max_ and AUC_tau_ were approximately 0.96 and 1.06, respectively, in the single-weekly dosing group and 0.76 and 0.93, respectively, for C_max_ and AUC_tau_ in the three-weekly dosing group. No significant accumulation of serum inetetamab was observed with either dosing regimen. The peak and trough concentrations of inetetamab in the single-weekly dosing group and the three-weekly dosing group reached or approached a steady state after approximately the 2nd dose (Figure 3), with mean peak concentrations of 68.1 μg/mL and 160.3 μg/mL, respectively; the mean trough concentrations were 26.5 μg/mL and 13.3 μg/mL, respectively.

### Treatment efficacy

Both BOR and unconfirmed BOR were observed in 22 (40.7%) of the 54 patients in the FAS set at 24 weeks, accounting for 40.7% of the total. Among these patients, 11 (40.7%) were in the single-weekly dosing group, whereas the remaining 11 patients (40.7%) were in the three-weekly dosing group (Table 4). A confirmed DCR at 24 weeks was observed in 46 patients, representing 85.2% of the patients. Among them, 24 patients (88.9%) were in the single-weekly dosing group, and 22 patients (81.5%) were in the threeweekly dosing group. The *P* values of 24-week BOR, 24-week unconfirmed BOR and 24-week confirmed DCR in the single-weekly dosing group and three-weekly dosing group were 1.000, 1.000 and 0.704, respectively, indicating that there was no statistically significant difference in the efficacy indices between the two groups.

**Table 4:** Efficacy endpoint (24-week) analysis (FAS)

	Single-week administration group (*n* = 27)	Three-week administration administration group (*n*=27)	*P*
BOR rate			1.000^a^
Occurred	11 (40.7)	11 (40.7)	
Not occurred	16 (59.3)	16 (59.3)	
unconfirmed BOR			1.000^a^
Occurred	11 (40.7)	11 (40.7)	
Not occurred	16 (59.3)	16 (59.3)	
Disease control rate			0.704^b^
Occurred	24 (88.9)	22 (81.5)	
Not occurred	3 (11.1)	5 (18.5)	

^a^ Chi-square test; b: Fisher test; BOR, best overall response.

### Immunogenicity

An analysis of the immunogenicity test results of the 57 patients included in the SS set revealed that none of the patients tested positive for ADA in this study. Consequently, titers and Nab levels were not assessed. The overall incidence of ADA was 0 in all the dose groups both before and after multiple infusions of inetetamab in patients.

### Treatment safety

A total of 594 AEs occurred in 27 patients (100%) in the SS single-weekly dosing group (Table 5), 9 SAEs in 7 patients (25.9%), 587 adverse reactions to inetetamab in 27 patients (100%), 311 AEs related to inetetamab in 24 patients (88.9%), 546 AEs related to vinorelbine injection in 26 patients (96.3%), and 2 AEs leading to discontinuation in 1 patient (3.7%).

**Table 5:** Summary of adverse events (AE)

	Single-week administration group (*n*=27)			Three-week administration group (*n*=30)			*P*
		
	Time	Number of subjects	Incidence rate (%)	Time	Number of subjects	Incidence rate (%)		
AE	594	27	100	553	30	100	1.00
SAE	9	7	25.9	18	10	33.3	0.54
ADR	587	27	100	542	28	93.3	0.49
AE associated with inetetamab	311	24	88.9	372	25	83.3	0.71
AE associated with vinorelbine injection	546	26	96.3	527	29	96.7	1.00
AE that led to drug withdrawal	2	1	3.7	4	3	10	0.61
ADR that resulted in discontinuation	0	0	0	3	2	6.7	0.49
AE that caused death	0	0	0	1	1	3.3	1.00
AE leading to withdrawal from the trial	0	0	0	2	2	6.7	0.49

AE: adverse event; SAE: server adverse event; ADR: adverse drug reactions

There were a total of 553 AEs in 30 patients (100%) in the three-weekly dosing group, 18 SAEs in 10 patients (33.3%), 542 inetetamab adverse reactions in 28 patients (93.3%), 372 inetetamab-associated AEs in 25 patients (83.3%), 29 patients (96.7%) had a total of 527 vinorelbine injection-related AEs, and 3 patients (10%) had a total of 4 AEs leading to discontinuation. ADRs leading to discontinuation (inetetamab ADR), AEs leading to death, and AEs leading to withdrawal from the trial occurred in the three-weekly dosing group. They occurred in 2 patients (6.7%) on a total of 3 occasions, 1 patient (3.3%) on a total of 1 occasion and 2 patients (6.7%) on a total of 2 occasions. One of the AEs leading to death was not related to inetetamab, one of the AEs leading to withdrawal was a fatal AE not related to inetetamab, and the other was likely not related to inetetamab.

In the single-weekly dosing group, grade 1 AEs occurred in 1 patient (3.7%) for a total of 295 occurrences (49.7%), grade 2 AEs occurred in 8 patients (29.6%) for a total of 214 occurrences (36.0%), and grade ≥3 AEs occurred in 18 patients (66.7%) for a total of 85 occurrences (14.3%). The three-weekly dosing group experienced grade 1 AEs in 3 patients (10.0%) for a total of 282 events, grade 2 AEs in 7 patients (23.3%) for a total of 167 events, and grade ≥3 AEs in 20 patients (66.7%) for a total of 104 events. Notably, one unintended serious adverse reaction (hypotension) occurred in one patient in the three-weekly dosing group.

## Discussion

Prior clinical trials have demonstrated that inetetamab, the active component of cipterbin, has efficacy and safety comparable to those of trastuzumab. These findings underscore its significance and potential as a first-line treatment, offering a novel targeted therapeutic option for patients with HER2+ metastatic breast cancer. Thepharmacokinetic properties and therapeutic efficacy of weekly administration of inetetamab have been evaluated in previous clinical investigations. For the administration of inetetamab, previous studies have suggested a weekly dosing regimen. However, this administration method is often difficult for patients to accept and increases the number of hospitalizations due to the short dosing interval and lack of coordination with the dosing time of chemotherapy drugs. Therefore, it is particularly important to appropriately extend the dosing interval of inetetamab while ensuring the effective drug concentration, and to make the dosing time of inetetamab and chemotherapy drugs close to each other for the convenience of patients. Thus, this study aimed to improve the mode of administration. Specifically, the pharmacokinetic profile, safety, efficacy, and immunogenicity of inetetamab in combination with vinorelbine injection were compared in both single-weekly and three-weekly regimens.

In terms of pharmacokinetics, our findings indicate that, following the initial intravenous drip of inetetamab, patients exhibited similar median T_max_ values in both the single-weekly dosing group and the three-weekly dosing group, a nearly linear increase in C_max_ with increasing dose, and an AUC increase that exceeded the dose increase, and a tendency for an increase in t_1/2_ with increasing dose. However, t_½_ could not be accurately determined in the single-weekly dosing group after the 7th and final doses because the AUC__%Extrap_ exceeded 20% or some patients did not complete the intensive sampling cycle. No significant increase in t_½_ was observed for inetetamab as the number of doses increased in the three-weekly dosing group. No significant accumulation of inetetamab was observed in the serum of either treatment group. Inetetamab trough concentrations all reached or approached a steady state approximately after the 2nd dose, with mean C_max_, C_min_, AUC_tau_, and C_av_ values in the three-weekly dosing group approximately 2-fold, 0.42-fold, 3-fold, and 1-fold greater than those in the single-weekly dosing group, respectively. The mean steady-state concentrations of C_av_ were comparable between the two dosing regimens.

In terms of efficacy, there were no significant differences in 24-week BOR, 24-week unconfirmed BOR, 24-week confirmed DCR, or OS between the single-weekly dosinggroup and the three-weekly dosing group, suggesting that the change in the mode of administration of inetetamab in combination with vinorelbine injection had little effect on efficacy, and both groups indicated favorable efficacy in this group of patients. TRAVIOTA is a phase II clinical trial comparing the efficacy and tolerability of trastuzumab in combination with vinorelbine or paclitaxel-based chemotherapy in the first-line treatment of patients with HER2-overexpressing metastatic breast cancer.^[29]^ In the vinorelbine combined with trastuzumab arm of the TRAVIOTA study, patients who had not received prior chemotherapy for advanced disease received trastuzumab at an initial loading dose of 2 mg/kg followed by weekly administration of 4 mg/kg combined with vinorelbine at 25 mg/m^2^ per week, with an overall remission rate (CR + PR) of 51%. Owing to significant differences in sample size and study design, our results cannot be directly compared with previous data.

In addition, the ADA positivity rate was 0 in all 57 patients in the SS set and did not increase with increasing dose or number of doses administered in each dose group. This finding shows that the immunogenicity of the drug in this trial did not affect its effectiveness or safety. However, a study comparing the incidence of heart failure among different treatment regimens for HER2+ breast cancer patients based on World Health Organization pharmacovigilance data revealed that 41, 976 patients had ADRs with anti-HER2 monoclonal antibodies (trastuzumab, *n* = 16, 900; pertuzumab, *n* = 1, 856), antibody-drug conjugates (trastuzumab emtansine [T-DM1], *n* = 3, 983; trastuzumab deruxtecan, *n* = 947), and tyrosine kinase inhibitors (TKIs, afatinib, *n* = 10, 424; lapatinib, *n* = 5, 704; neratinib, *n* = 1, 507; tucatinib, *n* = 655); additionally, 36, 052 patients had ADRs with anti-HER2-based combination regimens.^[30]^ The positive rate of ADA of 0 may be related to the smaller number of subjects in our study, and the sample size should be increased in the future.

The incidences of AEs, SAEs, AEs of inetetamab, AEsrelated to inetetamab, AEs related to vinorelbine injection, and AEs leading to discontinuation were similar in the single-weekly dosing group and the three-weekly dosing group, and the incidence of AEs at all levels was generally consistent between the groups. Although ADRs leading to discontinuation (inetetamab ADR), AEs leading to death, and AEs leading to withdrawal from the trial occurred only in the three-weekly dosing group, ADRs leading to discontinuation occurred only in 2 patients (6.7%), for a total of 3 instances. One of the AEs that resulted in death leading to patient withdrawal was not related to inetetamab, and the other AE that resulted in withdrawal was likely not related to inetetamab. As a result, there was no significant difference in the safety and tolerability of inetetamab in combination with vinorelbine injection between the single-weekly and three-weekly dosing groups. However, one unintended serious AE (hypotension) occurred in one patient in the three-weekly dosing group, and attention should be given to such AEs during subsequent dosing regimens, with prompt symptomatic treatment. In terms of cardiotoxicity, inetetamab performed better than trastuzumab and anthracyclines. Many studies have shown that pairing anthracycline-based chemotherapy with trastuzumab can lead to significant AEs such as cardiotoxicity, which has led to a gradual shift from anthracycline-based regimens to paclitaxel-based regimens for the treatment of early-stage HER2+ breast cancer in the clinic.^[31]^ In a phase II trial of trastuzumab combined with vinorelbine for treating advanced HER2+ breast cancer in women, one of 68 subjects developed symptomatic heart failure. This condition was effectively detected by a monitoring algorithm that screens for left ventricular ejection fraction (LVEF) at two weeks, but it remains a significant concern for future treatments.^[32]^ Intervention in the context of cardiotoxic adverse events caused by these drugs should receive increased attention. In thecase of inability to stop the drug or change the drug, the drugs that can be used to nourish the myocardium include coenzyme Q10, inosine, *etc*., to promote the metabolism of cardiomyocytes and help myocardial injury return to normal.^[33]^ Second, symptomatic treatment was carried out for cardiotoxic adverse events. Antiarrhythmic drugs are used if arrhythmia occurs. When heart failure occurs, strong heart, diuretic, and vasodilator drugs and other drugs are needed to improve heart function and reduce the burden on the heart.^[34]^ In a single-institution clinical experience, the combination of inetetamab and pyrotinib with vinorelbine demonstrates promising efficacy and safety as a second-line therapy and beyond for HER2-positive metastatic breast cancer, warranting further investigation into its potential role in the treatment landscape.^[35]^ Thus, this combination is an effective and safe alternative to trastuzumab and vinorelbine for the first-line treatment of HER2-positive locally advanced or metastatic breast cancer.

Our pharmacokinetic and safety data suggest that dosing at longer intervals may be feasible. Three-weekly dosing may provide additional benefits in terms of patient convenience, cost of treatment, and reduced impact on drug efficacy and occurrence of AEs.^[33]^ The strengths of this study, compared with those of previous studies, include altering the mode of administration and investigating the pharmacokinetic profile, safety, efficacy, and immunogenicity of the anti-HER2 innovator inetetamab in combination with vinorelbine in HER2+ patients with metastatic breast cancer who have received one or more chemotherapy regimens. The small sample size of subjects included was a drawback of this study; therefore, more rigorous conclusions were not possible. All patients who had received one or more chemotherapy regimens with or without prior radiotherapy and surgery were included. This subgroup analysis may allow for more accurate pharmacokinetic testing of the combination of different modes of administration. In addition, although the three-weekly and single-weekly dosing regimens had similar efficacy and safety profiles, it is possible that other dosing regimens may be more aggressive in patients with life-threatening illnesses. Another limitation of this study is that, owing to the geographical limitations of the study patients, the universality of the study across different ethnicities needs to be discussed; thus, more international multicenter clinical studies are needed. Follow-up studies should be conducted to explore the true effect of inetetamab in combination with vinorelbine as first-line therapy.

In summary, our findings indicate that the combination of inetetamab and vinorelbine administered every three weeks resulted in slight differences in the mean C_max_, C_min_, AUC_tau_, and C_av_ compared with those in the single-weeklydose group. However, the mean steady-state concentration of C_av_ was comparable between the two groups. Moreover, in patients with HER2+ metastatic breast cancer, the use of inetetamab every three weeks in combination with vinorelbine was found to be safe and well tolerated, with efficacy falling within the expected range. The incidence and severity of AEs and adverse reactions were similar compared to those of the single-weekly dosing regimen. Consequently, a three-weekly inetetamab regimen could serve as a viable substitute for weekly dosing. The outcomes of our trial provide support for further studies of inetetamab every three weeks in combination with vinorelbine regimens, offering patients a more convenient and cost-effective dosing schedule. Compared with the single-weekly dosing regimen, the three-weekly dosing regimen can reduce the frequency of patients traveling to the hospital for treatment and the total number of days in the hospital, freeing bed resources. This means that the cost of medical treatment for patients will be greatly reduced, and limited medical resources will be able to serve more patients.
